# Quicker team launch times for urgent priority neonatal retrievals: A Quality Improvement Initiative study

**DOI:** 10.1038/s41372-025-02354-6

**Published:** 2025-07-23

**Authors:** Saumil Desai, Kevin George, Kylie McDonald, Alysha Timoney, Dahna Kelland, Stephanie Barr, Molly Carroll, David Lockhart, Olivia Peters, Matt Cooper, Jonathan Davis

**Affiliations:** 1 Newborn Emergency Transport Services (NETS), Perth Children’s Hospital, Nedlands, WA Australia; 2 Neonatal Intensive Care Unit, Perth Children’s Hospital, Nedlands, WA Australia; 3 School of Medicine, The University of Western Australia, Crawley, WA Australia; 4 Neonatal Intensive Care Unit, King Edward Memorial Hospital, Subiaco, WA Australia; 5 Department of Biostatistics, The Kids Research Institute Australia, Nedlands, WA Australia

**Keywords:** Paediatrics, Outcomes research

## Abstract

**Background:**

Neonatal retrieval networks have adopted time-centric quality metrics as Key Performance Indicators (KPI) for setting and comparing benchmarking standards. Quicker launch time (departure from base), an essential KPI, enables neonatal retrieval teams to rapidly provide higher-level care to sick infants. The Newborn Emergency Transport Services of Western Australia (NETS WA) facilitates neonatal transfers across largest global retrieval area necessitating quicker team launch times for urgent retrievals. NETS WA conducted a quality improvement (QI) study to quicken team launch times for urgent retrievals.

**Aims:**

The smart aim was to quicken NETS WA team launch times on urgent retrievals to comply with the recent Australian New Zealand Neonatal Retrieval Network 2022 benchmark ( < 15 min). Secondary aims included impact of quicker launch times on “first look time” (time from decision to retrieve to presence at bed side) and “total retrieval time” (total time taken from the decision to transport until the handover of the patient at the receiving hospital).

**Settings:**

This study was completed over two years in NETS WA. Urgent priority retrievals are 10–15% of total transfers (120–180/year).

**Interventions:**

Plan-Do-Study-Action (PDSA) cycles: 1. Immediate access to transport cots 2. Additional personnel 3. Pre-defined priority matrix 4. Direct communication strategies. Launch time, first look time and total retrieval time were gathered from an electronic retrieval database (REDCap). Data collection were done at baseline (January–May 2022), during PDSA cycles (June 2022–April 2023) and after last PDSA cycle (May–December 2023).

**Results:**

Times are expressed as median (interquartile range IQR) in minutes. Comparisons were made for all transports and for road and air transports separately. Launch times decreased from 35.5 (21.5–90.0) at baseline to 17.0 (11.0–37.0) minutes (p 0.0006) after the last PDSA cycle for all urgent priority retrievals. Launch times for road only decreased to 15.0 (10.0–20.0) minutes (p 0.009). First look time decreased from 85.0 (54.8–269.3) to 52.5 (30.5–152.3) minutes (p 0.008). Total retrieval time changed from 243.5 (135.8–395.3) to 182.0 (117.0–390.0) minutes (p 0.33).

**Conclusion:**

Well-designed QI measures enabled NETS WA teams to quicken essential time-centric quality metrics for urgent priority neonatal retrievals.

## Introduction

Centralization of tertiary neonatal services require retrieval teams to provide higher level care and interhospital transfer to sick or premature infants in distant settings [1]. Eight to 25% of neonatal retrievals with an imminent threat to life or organ are time-critical [2, 3]. These retrievals necessitate transport teams to attend as rapidly as possible. Delays in departure can impact the provision of this time-critical care [4].

Retrieval team departure time is called “mobilization time” by Ground and Air Medical Quality Transport (GAMUT 2015) database and “launch time” by the Australian New Zealand Neonatal Retrieval Network (ANZNRN 2022) data dictionary. This is an important time-centric quality metric indicator for benchmarking of retrieval teams [5–7]. Quicker launch times (“time taken from dispatch to leave base”) prove more beneficial than minimizing stabilization times (“time from first look at baby till ready to leave”) for urgent neonatal retrievals [4, 7, 8]. This is due to unique neonatal physiology requiring longer stabilization compared to adult population [9]. Launch times are influenced by several factors such as availability of a trained retrieval team and ambulance, triaging, equipment readiness and communication [4, 10].

The Newborn Emergency Transport Services, of Western Australia (NETS WA) is the sole retrieval service for transferring neonatal infants across the state. WA is the largest retrieval area in the world operated by a single team (2.6 million sq kms) [11–13]. NETS WA has prolonged retrieval durations and quicker launch times allow sick infants to be treated in a more time-critical manner. The present study aims to introduce quality improvement (QI) initiatives to quicken the NETS WA team launch times on urgent priority retrievals.

## Methods

Study setting: WA has approximately 34,000 live births annually [11],1100–1200 [12, 13] infants are transferred by NETS WA to one of two tertiary NICUs in the state capital, Perth. The furthest trip is 2200 km and may take ~24 h to complete[12–14]. Each NETS WA retrieval team consists of doctors (senior registrar and/or neonatal consultant), a nurse (neonatal and transport trained) and an ambulance transport officer.

NETS WA team operates with support from St John’s Ambulance services for Perth metropolitan urban area that sprawls over 175 kms (road only). Approximately ~70% of the NETS WA retrievals are within the Perth metropolitan area. NETS WA is supported by the Royal Flying Doctor Services Western Operations (RFDS) for transfers outside the metropolitan area ( ~ 30%) that require rotary and fixed wing aircraft for retrievals (road and air) [12–14]. The referral and transport process of NETS WA retrievals is like other standard neonatal retrieval teams (Appendix 1).

### Ethics approval

Institutional approval (Governance Evidence Knowledge Outcomes, GEKO WA project number 50894) was obtained to conduct a quality improvement (QI) study to quicken launch times. This study was approved by the Safety and Quality Framework of the Child and Adolescent Health Service of Western Australia (Governance Evidence Knowledge Outcomes, GEKO WA project no 50894). The study was conducted using routinely collected data for service improvement and received a waiver of ethics for the inclusion of this de-identified data. Informed consent was not required. All methods were performed in accordance with the relevant institutional ethics committee guidelines and regulations.

The study followed the standard SQUIRE 2.0 guidelines [15].

### Design

Single service centre QI study undertaken by NETS WA.

### Definitions

Launch time is the “Time the team departed base” (ANZNRN data dictionary, (2022)) [7]. The dictionary defines the “urgent” retrievals as those that should have launch times of less than 15 min ie priority 1 (p1) retrievals. The “serious” retrievals are classified as those requiring launch times between 30 and 60 min (p2 retrievals) and those classified as “Not time critical” could be more than 60 min (p3 retrievals) [7]. The other metrics evaluated were “first look time” and “total retrieval time”. “First look time” is defined as “time the NETS team first saw the patient” and the “total retrieval time” is the “total time taken from the initial referral call till the retrieval was completed” (ANZNRN 2022) [7].

### Aims and goals

The primary aim was to quicken the launch times of the NETS WA teams for urgent transfers. The SMART (Specific, Measurable, Achievable, Relevant, and Time-Bound) goal was to decrease the launch times of the NETS WA teams for urgent retrievals to ≤15 min (recommended by ANZNRN 2022) over two years [7]. The secondary aim was to evaluate the impact of quicker team launch times on the “first look time” and “total retrieval time”.

Study period: The study was conducted over two years (January 2022–December 2023).

Data collection: A complete list of study variables is described in Appendix 2. These variables are routinely captured by the NETS WA team members in REDCap web application during each retrieval. Data were collected before, during and after the PDSA cycles in the study period.

### Quality Improvement (QI) team

A core NETS WA QI team consisted of the NETS WA medical director, a lead retrieval consultant, neonatal senior registrars (2 to 5 years’ experience), clinical nurse consultant and senior neonatal nurses.

### Baseline period (January–May 2022)

Focus group discussions (FGD) were conducted to review local audits and existing literature at baseline prior to the introduction of the QI interventions. The data collected in this period determined the time-centric metrics of NETS WA teams on urgent priority retrievals at baseline.

**Fig. 1:**
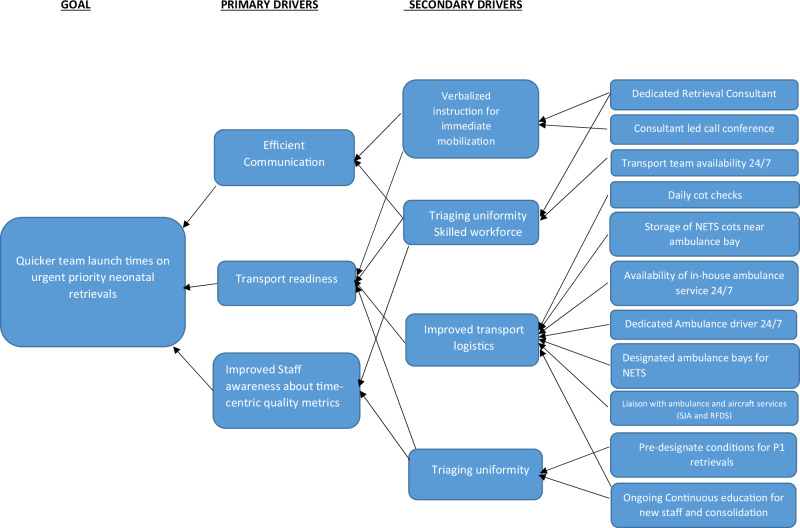
Driver diagram.

An online poll was conducted to explore factors impacting the current launch times. This delineated the root cause analysis and modifiable risk factors as described in the driver diagram (Fig. 1). Consensus-based QI initiatives were introduced as Plan-Do-Study-Action (PDSA) cycles in timely intervals to address these factors. PDSA cycles are a central component of QI initiatives and are impactful in neonatal care [16, 17]. Four PDSA cycles were introduced during the study period. FGD were organized at the beginning of each PDSA cycle and after the last PDSA cycle to ensure long term impact.

PDSA cycles and their timeline are detailed below:

#### PDSA cycle 1 (June 2022)

Equipment: Transport incubators (portable cot, ventilator, monitors, syringe pumps and transport fixation device) were moved and stored in a secure room next to the parked NETS ambulances. Prior to this the transport incubators were stored in the NETS WA office three floors above the ambulance departure area. The mobilization of NETS WA cots from level 3 to the ambulance for loading would take approximately 3–4 min. Decreasing this time was a key component of the first PDSA cycle.

Education: Weekly sessions to educate staff about NETS specific processes. Regular education sessions were conducted to orientate the new rotating staff about the definitions and expected standards for departure as per the ANZNRN data dictionary [7] (Appendix 2).

#### PDSA cycle 2 (October 2022)

Personnel: NETS WA referral calls were (until October 2022) led by the NICU consultants for management, in addition to their existing ward responsibilities. The newly recruited NETS WA only consultants were responsible for leading retrieval calls, management, and accompanying retrieval teams without any ward responsibilities. The QI team consolidated the NETS processes and education for time-centric quality metrics with this core group of dedicated neonatal retrieval consultants [7].

#### PDSA cycle 3 (February 2023)

Triage uniformity: A set of conditions (medical and surgical) were designated as automatic activation for P1 retrieval tasking of NETS WA teams from February 2023 (Appendix 3). This led to uniformity in tasking for P1 retrievals. Before this, P1 tasking was done at the discretion of the retrieval consultant.

Ongoing Education: Weekly sessions to educate new staff about NETS specific processes and time-centric quality metrics, as elaborated in PDSA cycle 1 (Appendix 2).

#### PDSA cycle 4 (April 2023)

Verbal Communication effectiveness: The need for urgent transfers were stated clearly by the retrieval consultant and then documented in the call conference notes. The team were also instructed to leave the call-conference and depart base within 15 min of this instruction.

Ongoing Education: Weekly sessions to educate new and rotating staff about NETS processes and time-centric quality metrics, as elaborated in PDSA cycle 1 (Appendix 2).

### After PDSA period (May–December 2023)

Data were collected for another 8 months to ensure improvement was sustained beyond the last PDSA cycle.

**Fig. 2:**
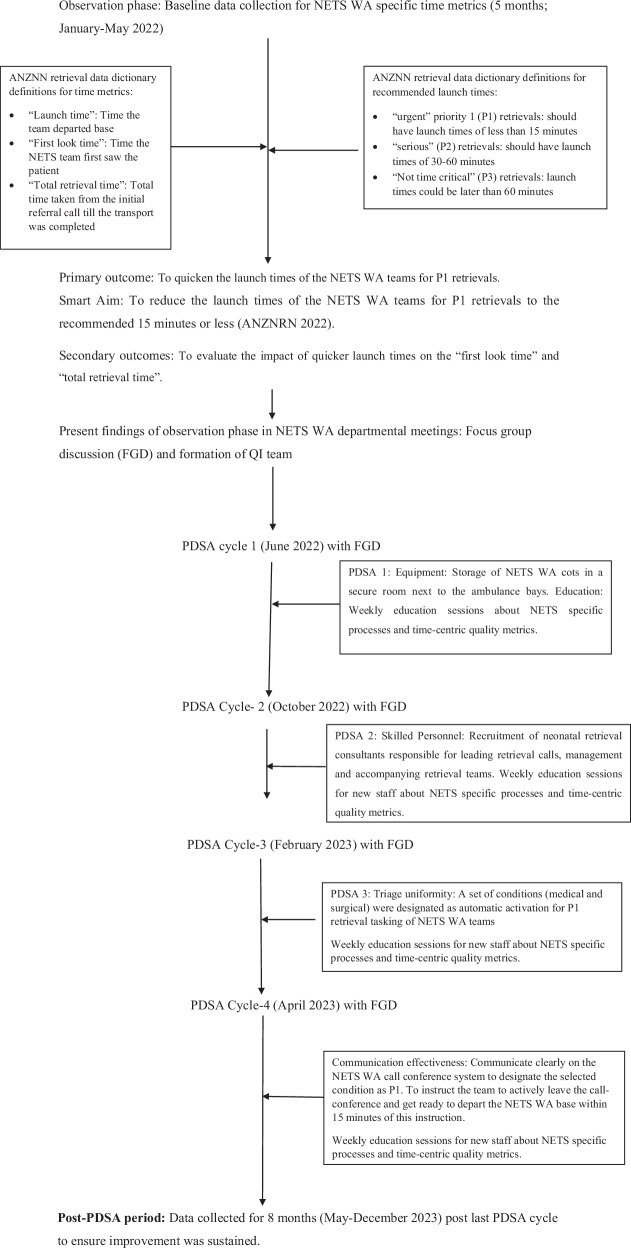
Study flow diagram.

The study flow chart with PDSA cycle timeline and data collection is depicted in Fig. 2.

### Statistical analysis

Data were compared for three epochs ie baseline: Jan–May 2022, PDSA period: June 2022–April 2023, after PDSA period May–December 2023.

Categorical variables were expressed using frequency, percentage and compared using the Fisher’s exact test. Continuous variables were expressed as median and inter-quartile ranges (IQR). Groups were compared using Kruskal-Wallis test with Dunn’s correction for multiple comparisons between the 3 epochs. A *p* value of <0.05 was considered statistically significant. Analysis was performed using GraphPad Prism (MA, USA).

The study period (January 2022–December 2023) was divided into monthly blocks for the run chart analysis. These describe median times of the time-centric quality metrics over the study period (Figs. 3.1–3.3). Common signals such as shift ( ≥ 6 consecutive points above or below the median line), trend ( ≥ 5 consecutive points increasing or decreasing) and astronomical data points (clear outliers due to extreme variation) were followed to interpret the run charts [18].

**Fig. 3:**
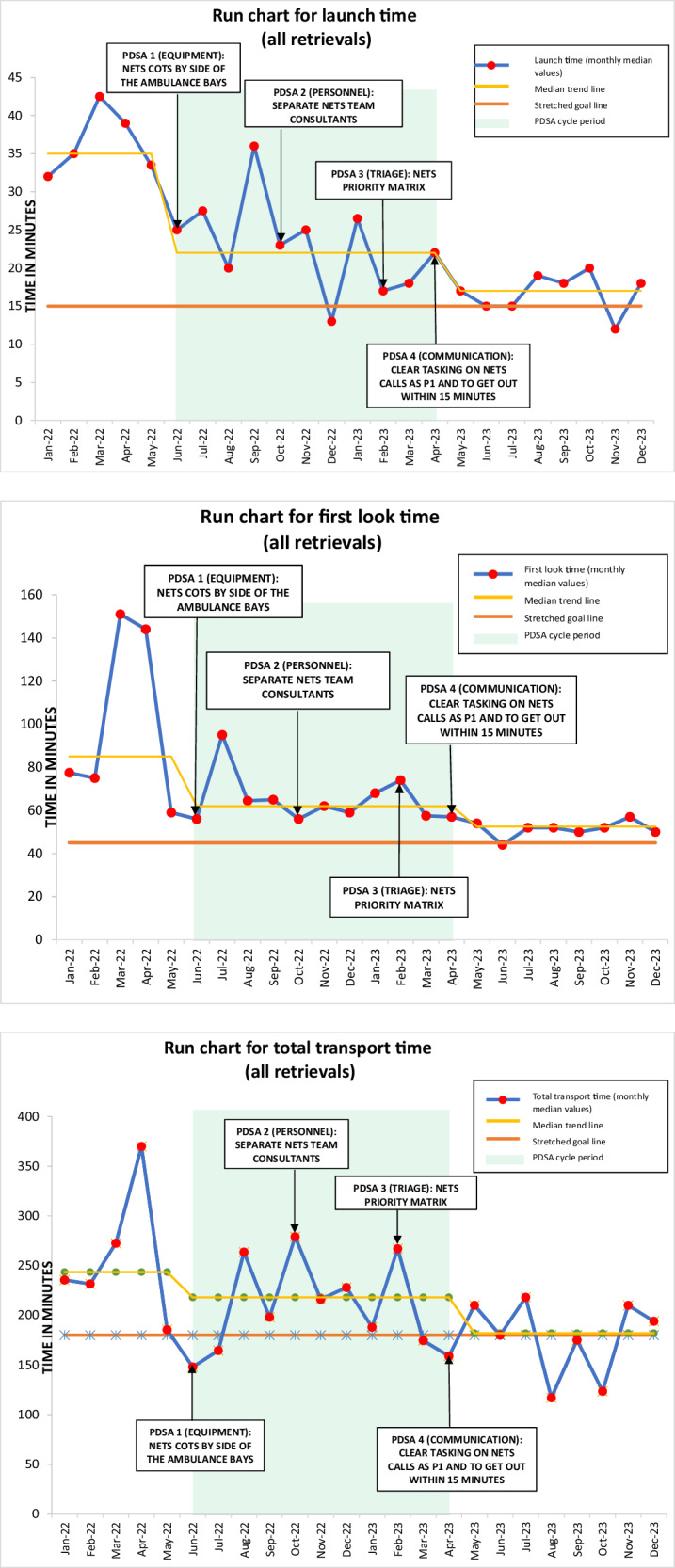
1: Run chart for launch time (all retrievals). 2: Run chart for first look time (all retrievals). 3: Run chart for total transport time (all retrievals).

## Results

NETS WA transported 1206 and 1120 retrievals in 2022 and 2023, respectively. The proportion of urgent retrievals increased from 110/1206 (9.12%) in 2022 to (172/1120 (15.35%)) in 2023 (*p* < 0.0001).

Comparisons were made for time-centric quality metrics (launch, first look and total retrieval time) for all transports (Table 1), road only (Table 2) and air transports (Table 3) separately. Times are expressed as median (interquartile range IQR) in minutes during baseline (Jan–May 2022), PDSA cycle period (Jun 2022–April 2023) and after PDSA cycle period (May–Dec 2023) (Tables 1–3).

**Table 1:** This table is for all (metro and regional) urgent priority NETS WA retrievals.

	January – May 2022 (1) (*n* = 42)	June 2022 –April 2023 (2) (*n* = 121)	May – December 2023 (3) (*n* = 119)	*p*
Gestational age at birth (completed weeks)	38.5 (34.7–40.2)	38.2 (32.5–39.4)	38.4 (35.1–40.0)	0.42
Birth weight (g)	3095 (2275–3493)	3000 (1964–3500)	3095 (2024–3513)	0.96
Retrieval reason				0.21
Non-Surgical	39	99	94	
Surgical	3	22	25	
P1 transfers achieving SMART aim of leaving base within 15 min *n* (%)	7 (16.66%)	46 (38.01%)	53 (44.53%)	**0.001**
Time taken to leave base. (minutes)	35.5 (21.5–90.0)	22.0 (13.0–45.0)	17.0 (11.0–37.0)	0.0006^*^
Time from decision to retrieve to first look. (minutes)	85.0 (54.8–269.3)	62.0 (40.0–170.5)	52.5 (30.5–152.3)	**0.008**^**§**^
Total time of transport (minutes)	243.5 (135.8–395.3)	218.0 (136.0–385.0)	182.0 (117.0–390.0)	0.33

^*^Significant *p* values in multiple comparisons: 1 vs. 2 = 0.02; 1 vs. 3 = 0.0004.

^§^Significant *p* values in multiple comparisons: 1 vs. 3 = 0.006.

Categorical data is presented as *n* (%) and compared across all groups by Fisher’s exact test. Continuous data is presented as median (IQR). Groups (1–3) were compared by non-parametric means (Kruskal-Wallis) with Dunn’s correction for multiple comparisons. Significant values are highlighted in bold. Additional significant comparisons are detailed below the table.

**Table 2:** This table is for urgent priority NETS WA retrievals needing only road (metro) transfers.

	January – May 2022 (1) *n* = 29	June 2022 –April 2023 (2) *n* = 84	May – December 2023 (3) *n* = 91	*p*
Gestational age at birth (completed weeks)	38.6 (35.1–40.1)	38.1 (31.6–39.4)	38.3 (35.6–40.0)	0.38
Birth weight (g)	3105 (2430–3485)	2995 (1813–3456)	3045 (2050–3500)	0.74
P1 transfers achieving SMART aim of leaving base within 15 min *n* (%)	7 (24.13%)	45 (53.57%)	51 (56.04%)	0.0028^#^
Time taken to leave base (minutes)	25.0 (16.0–35.0)	15.0 (12.0–25.0)	15.0 (10.0–20.0)	0.009*
Time from decision to retrieve to first look. (minutes)	58.0 (49.0–73.0)	45.0 (33.0–65.0)	44.5 (27.3–58.8)	0.03^§^
Total time of transport (minutes)	170.0 (112.5–204.5)	160.5 (114.8–235.8)	141.5 (104.0–211.0)	0.52

^#^Significant *p* values in multiple comparisons: 1 vs. 3 = 0.004.

^*^Significant *p* values in multiple comparisons: 1 vs. 3 = 0.007.

^§^Significant *p* values in multiple comparisons: 1 vs. 3 = 0.02.

Categorical data is presented as n (%) and compared across all groups by Fisher’s exact test. Continuous data is presented as median (IQR). Groups (1–3) were compared by non-parametric means (Kruskal-Wallis) with Dunn’s correction for multiple comparisons. Additional significant comparisons are detailed below the table.

**Table 3:** This table is for urgent priority NETS WA retrievals needing air (fixed wing/rotary wing aircraft) + road (regional) transfers.

	January – May 2022 (1) *n* = 13	June 2022 –April 2023 (2) *n* = 37	May – December 2023 (3) *n* = 28	*p*
Gestational age at birth (completed weeks)	39.5 (30.8–40.6)	38.9 (34–39.6)	39.0 (32.2–40.2)	0.8
Birth weight (g)	3090 (1418–3585)	3250 (2160–3865)	3180 (1998–3560)	0.4
P1 transfers achieving SMART aim of leaving base within 15 min *n* (%)	0 (0%)	1 (2.7%)	2 (7.14%)	0.32
Time taken to leave base. (minutes)	90 (57.5–152.5)	62.0 (36.5–145)	62.5 (24.3–131.3)	0.26
Time from decision to retrieve to first look. (minutes)	269.5 (202.8–318)	244 (171.5–394)	255 (185–345.5)	0.91
Total time of transport (minutes)	420 (370–512)	447 (385–518)	478 (388–602)	0.48

Categorical data is presented as *n* (%) and compared across all groups by Fisher’s exact test. Continuous data is presented as median (IQR). Groups (1–3) were compared by non-parametric means (Kruskal-Wallis) with Dunn’s correction for multiple comparisons. Significant values are highlighted in bold. Additional significant comparisons are detailed below the table.

Median launch time was 35.5 (21.5–90.0) minutes at baseline and decreased to 17.0 (11.0–37.0) (*p* = 0.0006) minutes after the PDSA cycle period for all urgent retrievals. Similarly, first look times were 85.0 (54.8–269.3) minutes at baseline and subsequently decreased to 52.5 (30.5–152.3) minutes (*p* = 0.008) after PDSA cycle period for all urgent retrievals (Table 1). A total of 7 (16.66%) out of 42 transfers in the baseline period met the SMART aim (launch time less than 15 min). The corresponding numbers were better with a total of 46 (38.01%) out of 121 transfers during the PDSA period meeting the SMART aim. There was further improvement in the post-PDSA period with a total of 53 (44.53%) transfers meeting the SMART aim. The difference in number of all transfers achieving the SMART aim between the baseline, during PDSA and after PDSA period was statistically significant (*p* = 0.001) (Table 1).

Median launch times for all road only (metro) urgent retrievals was in the recommended [7] duration of 15.0 (10.0–20.0) minutes (*p* = 0.009) in the post PDSA cycle period. Median (IQR) first look time was 58.0 (49.0–73.0) minutes at baseline and decreased to 44.5 (27.3-58.8) minutes (*p* = 0.03) after PDSA cycle period for all road only (metro) urgent retrievals. Total retrieval time was 170.0 (112.5-204.5) minutes and changed to 141.5 (104.0–211.0) minutes (*p* = 0.52) for all road only (metro) urgent retrievals (Table 2). The proportion of launch times that met recommendations increased for road only (metro) urgent transfers across all three epochs (7/29 (24.1%)) vs. 45/84 (53.6%) vs. 51/91 (56.0%); (*p* = 0.0028) (Table 2).

For air and road (regional) urgent priority retrievals launch times changed from 90.0 (57.5–152.5) minutes at baseline to 62.5 (24.3–131.3) minutes after PDSA cycles period (*p* = 0.26). First look time changed from 269.5 (202.8–318) minutes at baseline to 255 (185–345.5) minutes after PDSA cycle period (*p* = 0.91) (Table 3). The corresponding differences in number of rural retrievals achieving the SMART aim between the baseline, during PDSA and after PDSA period were not statistically significant (*p* = 0.32) (Table 3).

Run charts: Run charts depict the time metrics (launch, first look and total retrieval) for all urgent retrievals as median (IQR) (blue line with red dots) over monthly intervals in the study period (Fig. 3 1–3.3). The charts include a baseline period, PDSA cycle period (highlighted in green) and after PDSA period. The charts include an annual median line (yellow) of time metrics for both years in the study. They include a stretched goal line (orange) to indicate the desired goal. The charts show a downward shift in the time metrics from the baseline to after PDSA period towards the goal line. This suggests improvement with reduced durations for all the time metrics. There were 2 astronomical data points (1 each in baseline and during PDSA cycle period) (Fig. 3 1–3.3).

## Discussion

In the present QI study we have demonstrated the impact of well-planned PDSA cycles done by NETS WA team to improvise their time-centric quality metrics on urgent neonatal retrievals. These QI measures helped NETS WA team to achieve the desired “SMART goal” of quicker launch times for urgent priority metro retrievals to 15 min or less, recommended by the ANZNRN 2022 data dictionary (Tables 1, 2 Fig. 3) [7]. Quicker launch times were the most effective for road only retrievals within the metropolitan area, accounting for majority of the NETS WA retrievals ( ~ 70%) (Table 2, Fig. 3). Quicker launch times had a positive impact on the reduction of “first look time” and “total retrieval time”. Quicker first look time translated into NETS WA teams having earlier access to sick infants for provision of urgent time-critical care, lacking in those referring units. Reduced total retrieval time meant these sick infants received tertiary level care sooner (Tables 1–3, Fig. 3).

Neonatal transport networks, around the world have developed time-centric quality metrics for benchmarking. One metric unanimously endorsed is the team activation time referred to as mobilization or launch time [5–8]. Quicker launch times enable retrieval teams with rapid access to sick infants needing higher-level of care. Targeting quicker launch times is more practical than stabilization times in neonatal retrievals. This is due to the unique newborn physiology necessitating prolonged stabilization [9, 19]. Further, shorter stabilization times in adult and pediatric retrievals have failed to yield similar outcomes when extrapolated to the neonatal population [8, 20–22]. Hence, QI studies to achieve quicker launch times would be more beneficial in time-critical neonatal retrievals. However, it is challenging to perform QI studies in neonatal retrieval settings because of its dynamic nature.

There are only a few QI reports in the neonatal transport environment. Some recent effective QI measures in neonatal retrieval settings include reducing brain injury in extreme preterm infants and thermoregulation in outborn infants [23, 24]. There is a dearth of QI studies targeting launch times in urgent neonatal retrievals. Rajapreyar et al. reported an improvement in the mobilization times of their retrieval teams to less than 25 min. They did focused interventions such as recognizing the team with the quickest mobilization times and documenting reasons for delay [10]. The study involved road and rotary wing retrievals but no fixed wing aircraft retrievals. The study included both pediatric and neonatal retrievals with a mix of respiratory therapist or physician and a registered nurse [10]. In contrast, NETS WA have a more uniform approach of dispatching a senior neonatal registrar (advanced neonatal trainee) and/or consultant with a senior nurse for all urgent retrievals. Further, NETS WA transfers have a mix of road, fixed and rotary wing aircraft retrievals. Another QI study by Arcinue et al. targeted the mobilization time of their retrieval teams to less than 30 min [4]. Their PDSA cycles involved better communication skills, open critical bed space for transfers, faster patient room turnovers, more teams and involving the accepting neonatologist with the referring physician and transport physician on the initial call. The study showed success of these QI measures with the target mobilization time achieved in 82% of retrievals compared to 27% at baseline [4]. Kenningham et al. included 2 PDSA cycles with phase 1 involving separate dedicated retrieval team and phase 2 with process mapping. The study reported a 20% reduction of their mobilization time from baseline for road only transfers [25]. The present study differs from the above two studies as NETS WA focused on the “urgent” priority retrievals needing expedited launch times ( < 15 min) for the most time-critical conditions via road and/or air, over the largest retrieval area in the world. Further, the present study showed the impact of quicker launch times with an improvement in other essential time-centric quality metrics such as “first look time” and “total retrieval time”.

Our study has several strengths. It is the first conducted in an Australasian neonatal retrieval service and provides baseline data for recently agreed time-centric quality metrics (ANZNRN 2022) [7]. This may be helpful for national and international benchmarking. The SMART goal of targeting launch times (less than 15 min) was achieved within a reasonable time frame of two years. The impact of quicker launch times on improving other important time-centric quality metrics (first look time and total retrieval time) adds essential clinical relevance for urgent retrievals. Results were sustained over a long period after the last PDSA cycle. This adds credibility to the impact of the introduced QI measures. Study QI measures are generalizable and may be adapted by other neonatal retrieval services. The other strengths include standardized electronic data collection, regular FGD with education sessions and strict adherence to the SQUIRE 2.0 guidelines [15].

Limitations of our study was the lesser impact of the QI measures on non-metro retrievals. These retrievals are dependent on multiple factors such as availability of resources (aircraft, crew), aeromedical (pilot hours, civil aviation regulations) and weather constraints, that are outside of the control of the transport service. These are less amenable for any modifications. Another limitation was lack of assessment of impact of quicker launch times on scene/stabilization time. Reducing launch times is likely more beneficial than stabilization times for neonatal retrievals attributed to their unique physiology [9, 20, 21]. The lack of investigations around the astronomical data points and special cause variations in the run charts is another study limitation. We acknowledge another limitation in assessing the clinical outcomes and balancing measures of the improved time metrics during the study period. However, this is particularly challenging considering the heterogeneity of the neonatal conditions involved for urgent retrievals, different baseline characteristics and NICU courses. Clinical outcomes had little uniformity across this spectrum, and meaningful assessment of the impact of launch time on outcome was too difficult to ascertain. Further, we reported the impact of improved launch times on the “first look time” and “total retrieval time” showing an improvement. These improved time metrics can be considered as valid proxy measures for clinical outcomes. For example, improved first look time would translate into a specialist neonatal retrieval team assessing the sick infant earlier. Many of these infants potentially need medications (eg surfactant), procedures (eg intubation, chest drain) and management (eg ventilation, therapeutic hypothermia) that are not available at the referring hospital. Similarly, improved total retrieval times meant these sick infants were admitted to the tertiary neonatal units earlier. This potentially meant they could be assessed earlier by tertiary neonatologists and relevant sub-specialties (eg Surgical, Cardiology etc) for management. This could serve as a valid clinical proxy for outcomes such as time to initiate servo-controlled therapeutic hypothermia, need for high frequency ventilation, urgent cardiac scan for starting prostaglandin for duct dependent lesions and upper GI contrast studies for suspected malrotations to name a few (only available in tertiary neonatal units).

The future implications include regular PDSA cycles to ascertain the sustenance of these improved time metrics with investigations for the astronomical data points. There is scope for inter-agency collaboration, especially aeromedical transport services to improvise time metrics for distant retrievals. Studies could target the impact of improved time metrics in terms of clinical outcomes of retrievals involving infants with similar pathophysiological profile against a historical cohort. Further, it would be useful to compare these quality metrics with other ANZNRN and international neonatal retrieval services for benchmarking.

To conclude, well-designed QI measures enabled NETS WA teams to quicken essential time-centric quality metrics for urgent priority neonatal retrievals.

## Summary

### What is already known on this topic:

- Quicker launch times are crucial for retrieval teams for rapid access provision of higher-level care to sick neonatal infants.
- Published measures to decrease launch times of neonatal retrieval teams are lacking.

### What this study adds:

- Newborn Emergency Transport Services Western Australia (NETS WA) Quality Improvement (QI) measures decreased team launch times on urgent priority neonatal retrievals.
- Quicker launch times improved other essential time-centric quality metrics (“first look time” and “total retrieval time”) for urgent priority neonatal retrievals.

### How this study might affect research, practice or policy:

- The study provides data for consensus-based time-centric quality metrics for national and international benchmarking.
- Generalizable study QI measures for other neonatal retrieval teams to adopt and decrease their launch times.

## Supplementary information

SQUIRE checklist

Appendix 1-3 Information

## Data Availability

The data that support the findings of this study are available from the corresponding author upon reasonable request.
