# Supplementary material for: Quicker team launch times for urgent priority neonatal retrievals: A Quality Improvement Initiative study
Source: J Perinatol. 2025 Jul 23;46(1):93–101. doi: 10.1038/s41372-025-02354-6 (PMC12815656; doi:10.1038/s41372-025-02354-6)
Supplement: Supplementary file 1 — SQUIRE checklist [file 41372_2025_2354_MOESM1_ESM.docx]

| **Text Section and Item Name** | **Section or Item Description** | **Page number** |
| --- | --- | --- |
| Notes to authors | - The SQUIRE guidelines provide a framework for reporting new   knowledge about how to improve healthcare   - The SQUIRE guidelines are intended for reports that describe [system](#_bookmark13) level work to improve the quality, safety, and value of healthcare, and used methods to establish that observed outcomes were due to the [intervention(s).](#_bookmark8) - A range of approaches exists for improving healthcare. SQUIRE may be adapted for reporting any of these. - Authors should consider every SQUIRE item, but it may be inappropriate or unnecessary to include every SQUIRE element in a particular manuscript. - The SQUIRE Glossary contains definitions of many of the key words in SQUIRE. - The Explanation and Elaboration document provides specific examples of well-written SQUIRE items, and an in-depth explanation of each item.   Please cite SQUIRE when it is used to write a manuscript. |  |
| **Title and Abstract** | | |
| **1. Title** | Indicate that the manuscript concerns an [initiative](#_bookmark6) to improve healthcare (broadly defined to include the quality, safety, effectiveness, patient- centeredness, timeliness, cost, efficiency, and equity of healthcare) | Page 1, lines 1-2 |
| **2. Abstract** | 1. Provide adequate information to aid in searching and indexing 2. Summarize all key information from various sections of the text using the abstract format of the intended publication or a structured summary such as: background, local [problem,](#_bookmark10) methods, interventions,   results, conclusions | Page 3, lines 47-75 |
| **Introduction** | *Why did you start?* |  |
| [**3. Problem**](#_bookmark10) [**Description**](#_bookmark10) | Nature and significance of the local [problem](#_bookmark10) | Page 5, lines 113-118 |
| **4. Available knowledge** | Summary of what is currently known about the [problem,](#_bookmark10) including relevant previous studies | Page 5, lines 99-112 |
| **5.** [**Rationale**](#_bookmark12) | Informal or formal frameworks, models, concepts, and/or [theories](#_bookmark14) used to explain the [problem,](#_bookmark10) any reasons or [assumptions](#_bookmark0) that were used to develop the [intervention(s),](#_bookmark8) and reasons why the [intervention(s)](#_bookmark8) was  expected to work | Page 6, lines 151-156 |
| **6. Specific aims** | Purpose of the project and of this report | Page 6, lines 151-156 |
| **Methods** | *What did you do?* |  |
| **7.** [**Context**](#_bookmark1) | Contextual elements considered important at the outset of introducing the [intervention(s)](#_bookmark8) | Pages 7-8, Lines 161-207 |
| **8.** [**Intervention(s)**](#_bookmark8) | 1. Description of the [intervention(s)](#_bookmark8) in sufficient detail that others could reproduce it   Specifics of the team involved in the work | Page 7-8, Lines 161-207 |
| **9. Study of the Intervention(s)** | 1. Approach chosen for assessing the impact of the [intervention(s)](#_bookmark8)   Approach used to establish whether the observed outcomes were due to the [intervention(s)](#_bookmark8) | Pages 7-8, Lines 161-207 |
| **10. Measures** | 1. Measures chosen for studying [processes](#_bookmark11) and outcomes of the [intervention(s),](#_bookmark8) including rationale for choosing them, their operational definitions, and their validity and reliability 2. Description of the approach to the ongoing assessment of contextual elements that contributed to the success, failure, efficiency, and cost   Methods employed for assessing completeness and accuracy of data | Page 5, Lines 104-111 |
| **11. Analysis** | 1. Qualitative and quantitative methods used to draw [inferences](#_bookmark5) from the data 2. Methods for understanding variation within the data, including the   effects of time as a variable | Pages 8-9, Lines 209-221 |
| **12. Ethical**  **Considerations** | [Ethical aspects](#_bookmark2) of implementing and studying the [intervention(s)](#_bookmark8) and how they were addressed, including, but not limited to, formal ethics review and potential conflict(s) of interest | Page 6, Lines 132-140 |
| **Results** | *What did you find?* |  |
| **13. Results** | 1. Initial steps of the [intervention(s)](#_bookmark8) and their evolution over time (*e.g.*, time-line diagram, flow chart, or table), including modifications made to the intervention during the project 2. Details of the [process](#_bookmark11) measures and outcome 3. Contextual elements that interacted with the [intervention(s)](#_bookmark8) 4. Observed associations between outcomes, interventions, and relevant contextual elements 5. Unintended consequences such as unexpected benefits, problems, failures, or costs associated with the [intervention(s).](#_bookmark8)   Details about missing data | Pages 9-10, Lines 223-263 |
| **Discussion** | *What does it mean?* |  |
| **14. Summary** | 1. Key findings, including relevance to the [rationale](#_bookmark12) and specific aims   Particular strengths of the project | Page 11, Lines 266-276  Page 12, Lines 312-322 |
| **15. Interpretation** | 1. Nature of the association between the [intervention(s)](#_bookmark8) and the outcomes 2. Comparison of results with findings from other publications 3. Impact of the project on people and [systems](#_bookmark13) 4. Reasons for any differences between observed and anticipated outcomes, including the influence of [context](#_bookmark1)   Costs and strategic trade-offs, including [opportunity costs](#_bookmark9) | Pages 11-13 |
| **16. Limitations** | 1. Limits to the [generalizability](#_bookmark3) of the work 2. Factors that might have limited [internal validity](#_bookmark7) such as confounding, bias, or imprecision in the design, methods, measurement, or analysis   Efforts made to minimize and adjust for limitations | Pages 13-14, Lines 306-313 |
| **17. Conclusions** | 1. Usefulness of the work 2. Sustainability 3. Potential for spread to other [contexts](#_bookmark1) 4. Implications for practice and for further study in the field   Suggested next steps | Page 13, Lines 323-349 |
| **Other information** |  |  |
| **18. Funding** | Sources of funding that supported this work. Role, if any, of the funding organization in the design, implementation, interpretation, and reporting | Page 14 |
